# Commentary: PET/MR Versus PET/CT Imaging: Impact on the Clinical Management of Small-Bowel Crohn’s Disease

**DOI:** 10.3389/fmed.2017.00059

**Published:** 2017-05-24

**Authors:** Sophie James, Jonathan Tyrrell-Price

**Affiliations:** ^1^UH Bristol NHS Trust, Bristol, UK

**Keywords:** Crohn’s disease, fibrotic structures, strictures, MRI, PET imaging

Crohn’s disease is a chronic inflammatory condition affecting the gastrointestinal tract. A proportion of patients will suffer fibrotic narrowing in their inflammatory strictures. It is not currently possible to predict which patients will develop scarring, and no validated mechanism exists to detect the occurrence of fibrosis. Perhaps as a consequence, patients can present with obstructive symptoms after significant fibrotic damage has already accumulated (1).

The optimal treatment of Crohn’s disease has multiple objectives, but the avoidance of surgery is often a shared priority of both patients and health-care professionals. While medical options have unquestionably grown in recent years, fibrosis remains the physician’s Achilles heel, and consequently fibrostenosis remains the leading indication for surgery in small bowel disease (2, 3).

With no current medical treatment for existing fibrosis, early detection and intervention remain the physician’s best strategy; although symptoms are not always a sensitive guide. Once symptomatic stricturing has occurred, accurate differentiation of fibrosis from inflammatory strictures is key to appropriate surgical decision making. This scenario is further complicated by the mixed nature (inflammatory and fibrotic) of Crohn’s strictures (4, 5). Therefore, once stricturing has developed the fundamental clinical question is as follows: will this stricture respond to medical therapy? Accurate prediction of the therapeutic response would reduce unnecessary surgical interventions and shorten the time spent on futile immunosuppression.

It is at this latter point that Pellino et al. have compared PET/MR to PET/CT imaging for the detection of fibrosis in small bowel Crohn’s (6). The paper undertakes a comprehensive assessment of these imaging techniques in Crohn’s and their ability to provide data that alter surgical approach. It is how these methods predict the need to operate on symptomatic strictures, which will be focused on here.

Previous radiological studies have found that CT and MRI allow delineation of the internal morphology but inflammation frequently masks the fibrotic component of strictures. The addition of FDG PET allows assessment of metabolic activity and, therefore, better differentiation of fibrosis from inflammation. It is expected that fibrosis will generally be underdiagnosed and medication overused. This paper represents an attempt to describe the relative proportions of inflammation and scarring in strictures through radiological assessment compared to a surgical/pathological gold standard. Such studies could act as a precursor to assessing the medication sensitivity of strictures.

The provision of best possible care for the patients included in the study resulted in the findings at scan being used to inform therapy. Therefore, histological data from purely medically treated patients are not available, and only the medication responsiveness of patients felt to be “inflammatory” by imaging at study initiation (or who declined surgery) is known.

Thirty-five patients underwent both imaging tests on the same day. Eight patients went on to have an escalation in medical therapy for up to 2 weeks, seven of these eight were felt to have inflammatory-predominant disease. Two of these eight patients went on to require surgery. They were both found to have active inflammation and severe fibrosis histologically. PET/MRE detected the fibrosis in one patient (with that patient initially refusing surgery), whereas PET/CT missed the fibrosis in both cases. The six remaining medically treated patients were well at last follow-up (median 9 months, range 6–22 months). Of these 35 patients, at least 6 were medically treatable while at least 2 were not, 50% (1) were detected by PET/MRI versus none by PET/CT.

The focus of this paper was comparative detection of radiological findings against the gold standard of intraoperative assessment and histology; obviously requiring the patient to have surgery. The presence of a stricture was detected in 18 of the 21 narrowings found at surgery by both imaging modalities; PET/MRI distinguished the fibrotic component of 12 (57%) compared to 5 (24%) by PET/CT. This study was not structured to elucidate if these strictures would have responded to medical therapy. Where present the indication for surgery is likely to be the dominant stricture, it would, therefore, have been instructive to know if scans missed narrowings felt by the surgical team to be the source of any holdup.

There is no validated histological score for fibrosis and, therefore, no literature relating that score to medication responsiveness. The sensitivity of a stricture to medical therapy will likely be determined by the responsiveness of that patient’s immune system to that drug and the architecture of the stricture. A predominantly inflammatory stricture may prove resistant to an otherwise effective medication if it contains a tight band of fibrosis independent of the size of that band.

This paper shows a moderately increased sensitivity of MR/PET versus CT/PET and is, therefore, a valuable assessment of the radiological detection of fibrosis. However, while these imaging modalities are expensive and not available in all centers; its relevance remains largely academic.

Importantly, it shows the difficulty of accurately assessing patients at this crucial stage in their disease progression; even by expert teams with cutting edge scanning. Perhaps, this demonstrates the ethical acceptability of a medication first-based study as the fibrotic component cannot currently be accurately determined. Such a study could address the clinical question: “what is the likelihood of a patients strictures requiring surgery?”

Other groups assessed the potential of ultrasonography elasticity imaging (UEI) to evaluate for fibrosis in Crohn’s. UEI has been developed for use in other tissues, such as breast lesions and for liver fibrosis. There is evidence that ultrasound-derived stiffness measurements correlate with intestinal fibrosis assessed histologically and can differentiate fibrotic from inflamed bowel in *ex vivo* segments from patients with inflammatory bowel disease (7). Further small studies have shown that “real-time” UEI can accurately identify fibrotic sections of bowel (8, 9). In a study (8) of patients with established stenotic Crohn’s disease awaiting surgery, ultrasound-based measures of strain correlated highly between pre-, intra-, and post-operative values, which were similar to those values measured by direct tensiometry. UEI could potentially be developed into a safe, accessible, and non-invasive method to evaluate for fibrosis in Crohn’s patients. It is a promising technique; however, larger multicenter studies need to be performed before this could become a validated tool.

## Author Contributions

Both authors contributed equally.

## Conflict of Interest Statement

The authors declare that the research was conducted in the absence of any commercial or financial relationships that could be construed as a potential conflict of interest.
